# Retrospective Analysis of Spectrum of Presentation and Treatment Outcome in Extremity Sarcomas: A Single-Centre Experience

**DOI:** 10.1155/2018/4350634

**Published:** 2018-04-01

**Authors:** Saurabh Bansal, Kunal Das, Navneet Jain, Vipul Nautiyal, Meenu Gupta, Nadia Shirazi, Sanjiv Verma, Mushtaq Ahmad, Sunil Saini

**Affiliations:** ^1^Department of Radiotherapy, Cancer Research Institute, SRHU, Dehradun, Uttarakhand, India; ^2^Department of Medical Oncology & Hematology, Cancer Research Institute, SRHU, Dehradun, Uttarakhand, India; ^3^Department of Surgical Oncology, Cancer Research Institute, SRHU, Dehradun, Uttarakhand, India; ^4^Department of Pathology, Cancer Research Institute, SRHU, Dehradun, Uttarakhand, India; ^5^Department of Medical Oncology, Cancer Research Institute, SRHU, Dehradun, Uttarakhand, India

## Abstract

**Introduction:**

The most common site for soft tissue sarcoma is extremity. As complete surgical resection is possible in majority, outcome of this subset is relatively better. There is paucity of data regarding extremity soft tissue sarcoma (STS) from sub-Himalayan and hilly geographical regions.

**Materials and Methods:**

Retrospective analysis was done for extremity STS visiting the study center over a period of 5 years. Data were collected and analyzed for demography, disease characteristics, treatment modalities, and outcome.

**Result:**

Extremity STS constituted 32.8% of all STS enlisted. Most common subtype noted was pleomorphic STS. Metastatic disease at presentation was noted among 7/43 cases with lung being the most common metastasis site. Wide local excision was done in 37 cases while amputation was required in 5 cases. Adjuvant radiotherapy was given in 27 cases while 18 cases received adjuvant chemotherapy. At median follow-up of 47 months, the overall survival and event-free survival were noted as 47.64% and 41.49%, respectively.

**Conclusion:**

This study depicts single-center experience of extremity STS. The population analyzed was from sub-Himalayan region with significant lost to follow-up. Pooling of data from different centers has been advocated to derive conclusive results.

## 1. Introduction

Soft tissue sarcoma (STS) group of tumors are heterogeneous histologically distinct entities. It comprises around 1% of all malignancies in adult [1]. Extremities STS are the most common anatomical presentation of STS. The possibility of wide local excision and good local control makes it different from intraabdominal and head and neck STS. Surgery remains the mainstay of treatment supplemented by radiation therapy. While adjuvant radiotherapy is recommended for intermediate- and high-grade sarcomas, the role of adjuvant chemotherapy is still debatable. The data regarding STS clinicobiological behavior and outcome is sparse from sub-Himalayan region and similar high-altitude geographical regions. Even Indian data are sparse and limited to individual center experiences.

The Cancer Research Institute, Dehradun, is the largest and single referral tertiary cancer center in the state of Uttarakhand, India. We present our experience of soft tissue sarcoma occurring primarily in extremities.

## 2. Materials and Methods

A retrospective analysis was done for the patients diagnosed with extremities soft tissue sarcomas from January 2011 to December 2015. Electronic database of the hospital was searched for the patient details (maintained by the Uttarakhand State Council for Science and Technology support) and paper records were retrieved from medical record department. Institutional ethical committee clearance was taken beforehand. The details regarding the demographic profile of patients, anatomical and histological characteristics of STS, and treatment modalities and follow-up were collected. Records with insufficient or incomplete information regarding histology or treatment were excluded. Patients enrolled for second opinion were excluded, as no treatment modality was used. The outcome was assessed by electronic record of last visits and telephonic confirmation of survival or demise. Subsets that were not reachable telephonically were tried to contact by post.

Confirmation of diagnosis was done by core needle biopsy or incisional biopsy in all cases. All cases underwent staging workup with computer tomography (CT) of chest and involved limb magnetic resonance imaging. The 2013 WHO Classification for soft tissue sarcoma was used for tumor grouping. Sarcomas lacking characteristic histology, immunohistochemistry, and/or genetic features were categorized as unclassified/undifferentiated sarcoma [2]. Tumor grade was decided based on differentiation and mitotic figures and necrosis according to the Federation Nationale des Centers de Lutte Contre le Cancer (FNCLCC) system. Size, nodal status, and metastasis were noted as per AJCC staging system [3]. Surgical margin status was noted as clear or involved (microscopic or gross residual). A 6-week follow-up for initial two visits followed by 3 monthly visits for next 2 years was advocated to all cases as per institutional protocol. After 2 years of completion of therapy, a 6-month follow-up was planned for each case. Locoregional clinical examination was done at each visit, and annual imaging with CT chest was done. A relapse was confirmed histologically, and local as well as metastatic workup was done to define the pattern of relapse. Treatment-related toxicities requiring admission or intervention were recorded.

### 2.1. Statistical Analysis

The collected data was analyzed for demographic characteristics, tumor characteristics, and treatment modalities. Statistical analysis was done using SPSS version 17.0 (SPSS Inc., Chicago, IL). Time interval to local recurrence or distant failure was calculated from the date of start of treatment to date of recurrence detection, with censoring at date of death or last contact. Overall survival was calculated from treatment onset to date of death irrespective of the cause of death, with censoring at the date of last contact for the patient alive. Actuarial survival rates were calculated using the Kaplan–Meier method.

## 3. Result

During study period, a total of 250 cases of STS were enlisted. Out of which, 82 were STS of extremities. A cohort of 43 patients was analyzed as 39 cases either visited for opinion with diagnosis done outside or did not opt for surgical treatment in this center. Median age of presentation was 48 years (range 11–75 years), and there was noticeable male dominance (male : female = 28 : 15). Commonest histology noted was pleomorphic sarcoma (9/43), followed by synovial (7/43), and liposarcoma (6/43). Lower limb showed preferential site of occurrence in comparison to upper limb. Seven cases presented with metastatic disease with lung being the most common site (5/7). Two cases of each liposarcoma and synovial sarcoma and one case of leiomyosarcoma, malignant peripheral nerve sheath tumor, and pleomorphic STS each were metastatic at presentation. The median time lag of onset/observation of swelling or pain and diagnosis were 8.5 months. Postoperative grading and TNM was done in all cases. Nine cases were with tumor size <5 cm in maximal dimension. Majority (29/43) were high-grade histology while 4 were intermediate and 10 were low-grade sarcoma (Table 1).

Of 36 localized sarcomas, all underwent upfront surgical resection. Wide local excision was a preferred modality and was performed in 33 patients while 3 underwent limb amputation. Of 7 metastatic sarcomas, surgical resection was performed amongst 6 cases, all after first-line chemotherapy while 1 case opted for palliative care (Table 2). Three cases responded well to chemotherapy with disappearance of pulmonary metastasis, and two of them underwent amputation of limb while wide local excision was done in one. One case showed good partial response and underwent lung metastatectomy alone with wide local excision. Two cases showed mild reduction in size and underwent excision in view of local fungating mass over variable period of time. A total of 5 patients underwent amputation. Three were with neurovascular involvement rendering limb salvage difficult while two were with skip bone metastasis at the same limb. Wide local excision was performed in total of 37 cases (33 localized and 4 primary metastatic). Margin positivity was noted among 3 cases, only one underwent re-resection to achieve negative margin. One of them died later with recurrence of disease while other is alive till analysis. All 3 received adjuvant chemotherapy and radiotherapy to the local site. Postoperative complications noted amongst 3 cases in the form of wound dehiscence in two and partial graft necrosis in one case.

Radiotherapy was administered to 27 cases in adjuvant setting. Indication of radiotherapy involved surgical margin and/or intermediate to high grade of tumor. One case received upfront radiotherapy to primary and metastatic site as palliative care. In metastatic STS cohort, adjuvant radiotherapy was administered to 3 cases at primary site and 2 cases at metastasis site after good response to chemotherapy. The dose of radiotherapy was uniform to all 60–64 Gy in 2 Gy/day fractionation (external beam radiotherapy using linear accelerator with CT-based three dimensional conformal treatment planning), given in two phases. Phase I included tumor bed, postoperative scar, all drainage sites, and 3-4 cm margin in longitudinal plane and 1.5–2 cm margin in transverse plane with a dose of 44–46 Gy. Phase II was delivered for remaining dose on reduced field (tumor bed + 2 cm margin). Chemotherapy was administered to 6 metastatic cases upfront and 18 as adjuvant (ifosphamide-Adriamycin). One patient received low-dose chemotherapy with palliative goal (etoposide-Adriamycin).

**Figure 1:**
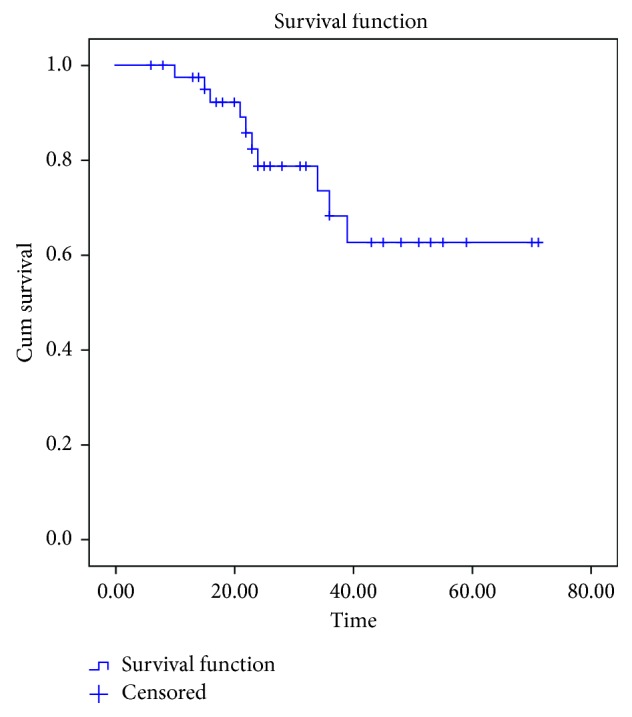
Kaplan–Meier curve showing overall survival (OS).

**Figure 2:**
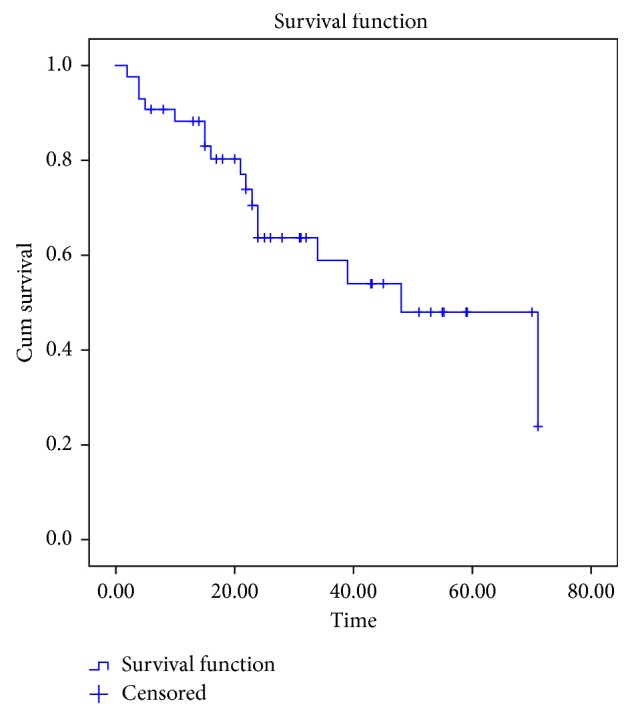
Kaplan–Meier curve showing event-free survival (EFS).

18 cases did not turn up for routine follow-up. Telephonic and postal communications resulted in noting outcomes among 5 cases; 13 cases could not be reached and were censored at last point of contact. Among 36 localized sarcomas, recurrence of disease was noted among 4 cases. Recurrence at primary site alone was noted among 3 cases and at lung alone in 1 case. Good locoregional control measures (surgery ± radiotherapy) resulted in low local recurrence. Of 7 primary metastatic cases, 1 opted for palliative care and died at 4 months. Of remaining 6 cases, three were surviving at the time of analysis, and 3 got recurrences (lung) and died later. With a median follow-up of 47 months (range 7–68 months), the overall survival and event-free survival were noted as 47.64% and 41.49%, respectively (Figures 1 and 2).

## 4. Discussion

Soft tissue sarcoma of adult encompasses wide histological variants. The most frequent sarcomas noted are liposarcoma, fibrosarcoma, and pleomorphic sarcoma. It can occur throughout the body; however, majority (60%) occurs in extremities [4, 5]. With reason unexplained, lower limb is a more preferred site of occurrence. Probably, large bulk of soft tissue at thigh and leg compartments is the reason. An unusual trend of early age presentation was noted in this study. Male sex has been reported more frequently with STS although this result is not consistent [6–8] The study group also noted 72% of STS occurrence in lower extremity and male predominance. However, in comparison to all sarcomas reported in center, limb sarcoma constituted only 32%. Cohort noted pleomorphic sarcoma as the most common variant followed by synovial sarcoma. The sample size is small to comment on incidence in general.

Diagnosis of STS of extremities is often delayed. Studies have tried to devise predictive clinical features for occurrence of STS. Swelling exceeding 5 cm, increase in size, pain, and deep location confer more chances of STS in the swelling [9]. Such swelling must be promptly examined by histopathology for STS. Fine-needle aspiration is not a preferred modality, and an excisional biopsy or punch biopsy should be done [10]. In this study, as standard protocol, all diagnosis was made on histopathology on tissues retrieved from incision or core needle biopsy and confirmed with immunohistochemistry in majority.

Surgical excision of tumor with wide margin, 4-5 cm to the sides and 1-2 cm deep to the tumor, is desired. Curative chances are majorly guided by a complete excision. If histopathology result shows narrow or positive margin, a resurgery to achieve a wide margin has clearly shown survival benefit over radiotherapy to margin [11, 12]. A re-resection was advised to cases with positive margin in this cohort; however, it was done in one case only. Re-resection was not technically feasible in one, and resurgery was declined by another. Amputation surgery has a very limited role in current era. In 90% of extremity STS, limb-sparing excision has been found feasible with local failure rate not exceeding 10% [13]. An infiltration of major blood vessel or nerve of limb practically makes limb salvage impossible. A plan for reconstruction with plastic surgery team is mandatory for amputation. Study cohort noted five cases of amputation, which happened in either nonsalvageable neurovascular involvement or locally recurrent STS-encasing blood vessel.

Role of adjuvant radiotherapy after a wide local excision is well established in high-grade and intermediate-grade STS. This multimodality achieves about 95% of local control; however, benefit in terms of overall survival is uncertain [14, 15]. Radiotherapy alone after a marginal resection is again inferior to reresection. The timing of radiotherapy is also controversial with no superiority of preoperative, or postoperative radiotherapy over another [16–18]. While preoperative radiotherapy makes tumor resectable, a chance of increased wound complications cannot be negated. Trials of perioperative radiotherapy with brachytherapy have been shown comparable to local control with limited radiation effects to nearby structures [19]. A neoadjuvant chemoradiotherapy followed by surgery and postoperative chemotherapy has shown comparable disease control and practically no negative impact of delay in surgery due to intensive neoadjuvant modality [20, 21]. Study cohort noted postoperative radiotherapy in 27 cases. It was given to either patient with involved margin or with intermediate-/high-grade feature. Radiotherapy was not given among low-grade tumors that underwent wide local excision, and margins were negative. Chemotherapy has been noted to have limited role except rhabdomyosarcoma and Ewing's group of sarcoma. Various trials have noted conflicting benefit of neoadjuvant/adjuvant chemotherapy with no benefit in overall survival [22, 23]. For locally advanced STS, reports of regional hyperthermia in combination of chemotherapy have shown better local control and outcome as well. Similarly, option of isolated limb perfusion of affected extremity with chemotherapeutic agents has been reported for primary inoperable limb sarcomas [24–26].

Extremities sarcoma has been noted to have better outcome, and the most likely explanation is complete surgical excision. Various studies have noted survival at 5 years as 65–75% with local recurrence rate of about 10% [27–29]. SEER data showed 3-year overall survival of 73% if radiotherapy was administered in adjuvant setting in high-risk STS [30]. Present study noted inferior overall and event-free survival. Censoring of cases at last follow-up and large number of lost to follow-up might be affecting the actual survival result. As this study analyzed population of hilly terrain, lost to follow-up was significant. In this study, telephonic and postal communication was used to ensure proper follow-up; however, a robust use of telemedicine and messaging by social networking would have been more useful. Study noted large number of high-risk STS which might have contributed to inferior outcome as well. This study presents the experience of extremity STS from a tertiary referral cancer center at sub-Himalayan region. The number of cases is small to draw any definitive conclusion regarding treatment efficacy or environmental effect on outcome. There is a need of pooling data from Himalayan/sub-Himalayan region to delineate effects of this geographical factor.

## Figures and Tables

**Table 1:** Tumor characteristics.

Tumor histology	Numbers
Pleomorphic sarcoma	9
Synovial sarcoma	7
Spindle cell sarcoma	5
Liposarcoma	6
Leiomyosarcoma	4
Fibrosarcoma	4
Dermatofibrosarcoma	3
Malignant peripheral nerve sheath tumor	2
Unclassified/undifferentiated	2
Epitheloid sarcoma	1
Grade	
Low grade	10
Intermediate grade	4
High grade	29
Location	
Upper limb	12
Lower limb	31
Size	
T1 (<5 cm)	9
T2 (>5 cm)	34
Metastasis	
M0	36
M1	7
Stage	
Stage I	10
Stage II	6
Stage III	20
Stage IV	7

**Table 2:** Treatment modalities.

Modalities	Nonmetastatic disease	Metastatic disease
Surgery		
Wide local excision	33	4
Amputation	3	2
Margin positivity	3	—
Chemotherapy		
First line	—	6
Adjuvant	18	4
Palliative	—	1
Radiotherapy	27	3
Relapse of disease/progressive disease	4	4
